# Crystal structures of the two salts 2-methyl-1*H*-imidazol-3-ium nitrate–2-methyl-1*H*-imidazole (1/1) and 2-methyl-1*H*-imidazol-3-ium nitrate

**DOI:** 10.1107/S2056989016003789

**Published:** 2016-03-11

**Authors:** Mouhamadou Birame Diop, Libasse Diop, Thierry Maris

**Affiliations:** a Laboratoire de Chimie Minérale et Analytique, Département de Chimie, Faculté des Sciences et Techniques, Université Cheikh Anta Diop, Dakar, Senegal; b Département de Chimie, Université de Montréal, 2900 Boulevard Édouard-Montpetit, Montréal, Québec, H3C 3J7, Canada

**Keywords:** crystal structure, 2-methyl-1*H*-imidazol-3-ium cations, bifurcated hydrogen bonds, chain structure

## Abstract

The title salts, C_4_H_7_N_2_^+^·NO_3_^−^·C_4_H_6_N_2_, (I), and C_4_H_7_N_2_^+^·NO_3_^−^, (II), were obtained from solutions containing 2-methyl­imidazole and nitric acid in different concentrations. In the crystal structure of salt (I), one of the –NH H atoms of the imidazole ring shows half-occupancy, hence only every second mol­ecule is in its cationic form. The nitrate anion in this structure lies on a twofold rotation axis. The neutral 2-methyl­imidazole mol­ecule and the 2-methyl-1*H*-imidazol-3-ium cation inter­act through N—H⋯N hydrogen bonds to form [(C_4_H_6_N_2_)⋯(C_4_H_7_N_2_)^+^] pairs. These pairs are linked with two nitrate anions on both sides through bifurcated N—H⋯(O,O) hydrogen bonds into chains running parallel to [001]. In the crystal structure of salt (II), the C_4_H_7_N_2_^+^ cation and the NO_3_^−^ anion are both located on a mirror plane, leading to a statistical disorder of the methyl H atoms. The cations and anions again inter­act through bifurcated N—H⋯(O,O) hydrogen bonds, giving rise to the formation of chains consisting of alternating anions and cations parallel to [100].

## Abstract 2

The title salts, C_4_H_7_N_2_^+^·NO_3_^−^·C_4_H_6_N_2_, (I), and C_4_H_7_N_2_^+^·NO_3_^−^, (II), are composed of hydrogen-bonded chains along [001] and [100] for (I) and (II), respectively.

## Chemical context   

While targeting the synthesis of new Sn^IV^ complexes, crystals of the salt C_4_H_7_N_2_^+^·NO_3_^−^, (II), were obtained serendipitously by mixing tri­methyl­tin acetate with 2-methyl­imidazole in the presence of nitric acid. In the dynamic of seeking new ammonium salts soluble in organic solvents that can be used for further metallorganic syntheses, we have initiated the targeted preparation of this salt. However, by variation of the ratio between nitric acid and 2-methyl­imidazole we also obtained crystals of compound (I), C_4_H_6_N_2_·C_4_H_7_N_2_^+^·NO_3_^−^, and report the two structures in this communication. 
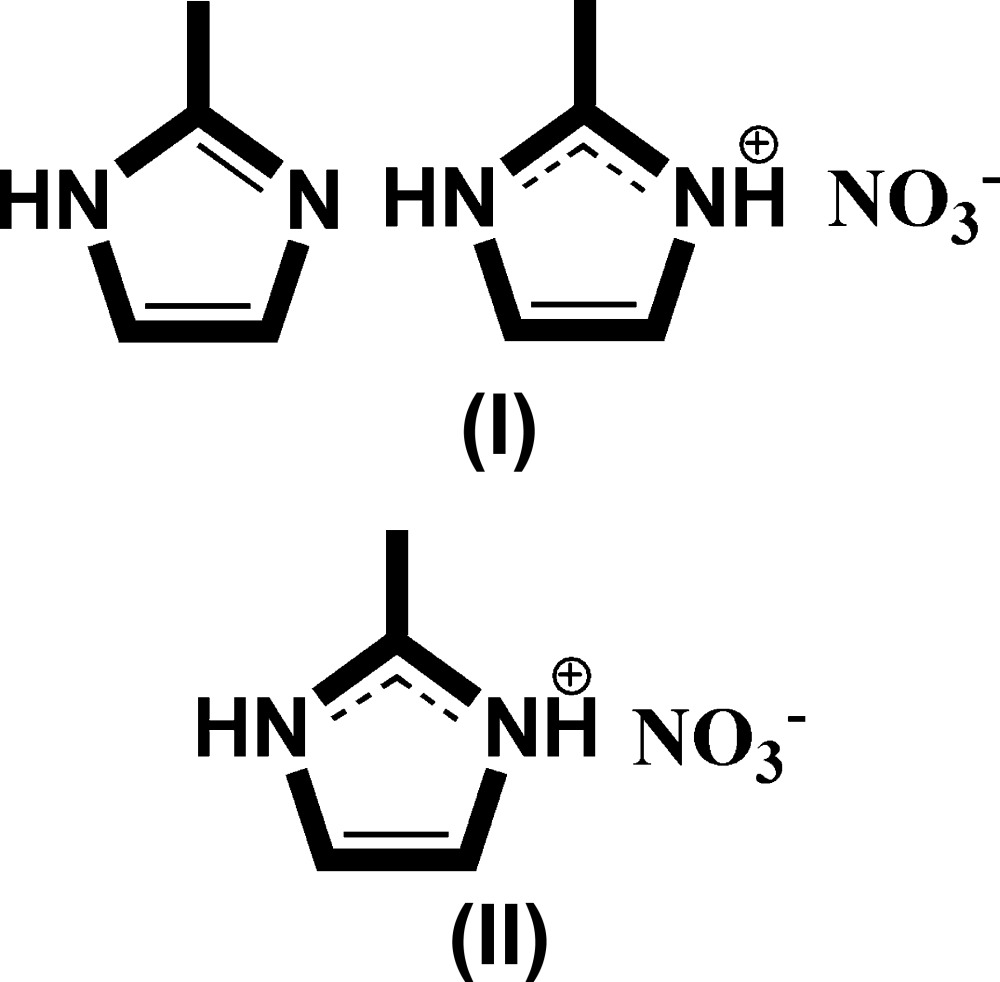


## Structural commentary   

**Figure 1:**
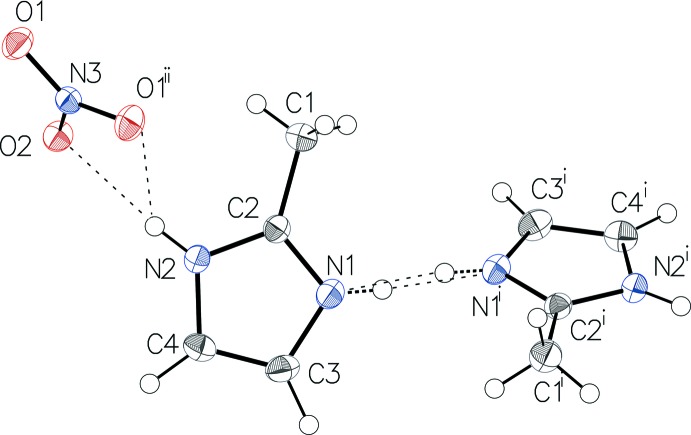
The mol­ecular components of salt (I), showing the atom labelling and displacement ellipsoids drawn at the 50% probability level. H atoms are drawn as spheres of arbitrary radius and hydrogen bonds are shown as dashed lines. [Symmetry codes: (i) 1 − *x*, *y*, 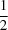 − *z*, (ii): 1 − *x*, *y*, 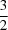 − *z*.]

**Figure 2:**
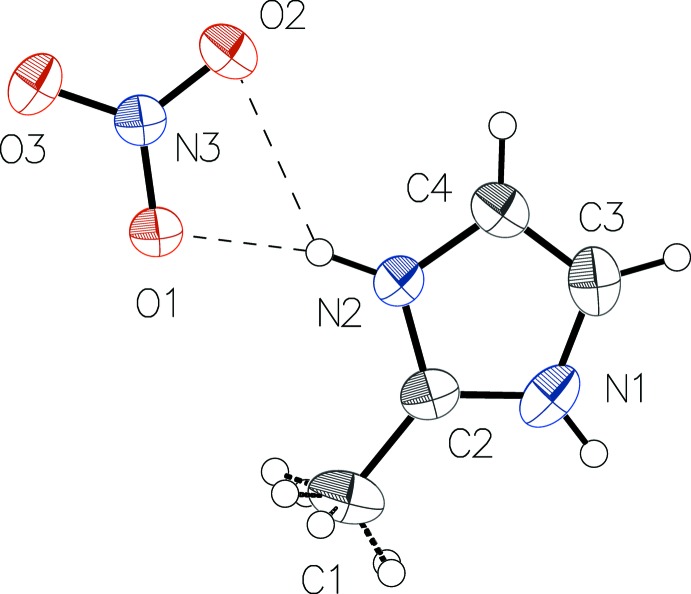
The mol­ecular components of salt (II), showing the atom labelling and displacement ellipsoids drawn at the 50% probability level. H atoms are drawn as spheres of arbitrary radius and hydrogen bonds are shown as dashed lines.

The asymmetric unit of salt (I) consists of a 2-methyl­imidazole moiety in a general position and part of a nitrate anion. The anion is completed by application of twofold rotation symmetry. The hydrogen atom H1 attached to N1 of the imidazole ring has a statistical occupancy of 0.5, thus leading to a 1:1 mixture of a 2-methyl-1*H*-imidazol-3-ium cation and a neutral 2-methyl­imidazole mol­ecule in the crystal applying symmetry operation (i) 1 − *x*, *y*, 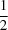 − *z* (Fig. 1 ▸). In the nitrate anion, the N—O bond lengths [1.2433 (11)–1.2774 (19) Å], are in a typical range (see, for example, Diop *et al.*, 2013 ▸) and indicate some π delocalization over the two oxygen atoms O1 and O1^i^. The longer N—O distance is observed for atom O2 involved in the stronger of the two observed N—H⋯O hydrogen bonds (Table 1 ▸). The imidazole ring is planar with a maximum deviation of 0.005 (1) Å. The asymmetric unit of salt (II) consists of an ordered 2-methyl-1*H*-imidazol-3-ium cation and a nitrate anion (Fig. 2 ▸), both lying on a mirror plane.

In the two structures, the O—N—O angles have normal values close to 120° and their sum (360°) reflect a perfect trigonal–planar geometry for each of the nitrate anions. For the 2-methyl-1*H*-imidazol-3-ium cations and for the neutral 2-methyl­imidazole mol­ecule, the N—C distances involving C2, the C atom that carries the methyl group, are equal within 0.01 Å, and their values are consistent with double-bond character, as previously observed (Diop *et al.*, 2015 ▸).

## Supra­molecular features   

**Figure 3:**
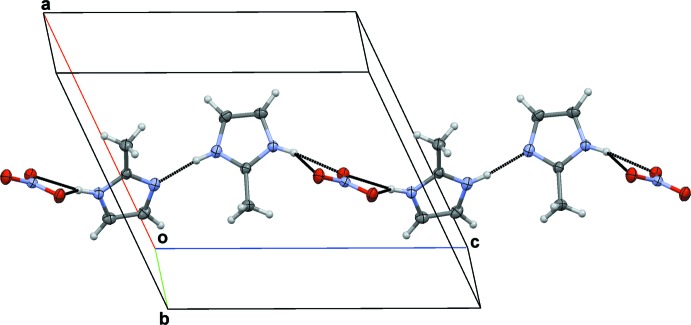
Partial view of the packing in the crystal structure of (I), showing a chain of hydrogen-bonded mol­ecules. Only one of the statistically disordered H-atom positions between the imidazole rings is shown.

In the crystal structure of salt (I), the neutral 2-methyl­imidazole mol­ecule is connected to the 2-methyl-1*H*-imidazol-3-ium cation through N—H⋯N hydrogen bonds, forming a [(C_4_H_6_N_2_)⋯(C_4_H_7_N_2_)^+^] pair (Fig. 1 ▸). Such pairs are then linked to two nitrate anions through bifurcated N—H⋯(O,O) hydrogen bonds (Table 1 ▸), leading to chains extending along [001] (Fig. 3 ▸).

**Figure 4:**
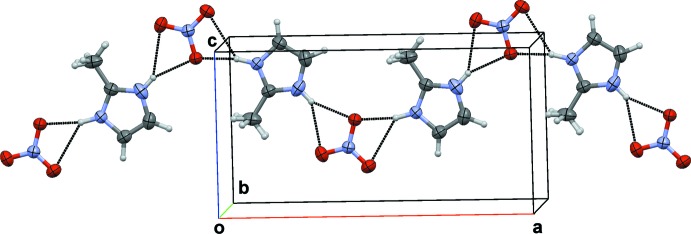
Partial view of the packing in the crystal structure of (II), showing a chain made up of hydrogen-bonded nitrate anions and 2-methyl-1*H*-imidazol-3-ium cations.

In the crystal structure of (II), the 2-methyl-1*H*-imidazol-3-ium cations and the nitrate anions are alternately linked by bifurcated N—H⋯(O,O) hydrogen bonds (Table 2 ▸), leading to the formation of hydrogen-bonded chains parallel to [100] (Fig. 4 ▸).

In the two structures, the stability between the chains is dominated by electrostatic inter­actions.

## Database survey   

A search of the Cambridge Structural Database (Version 5.37 with one update, Groom & Allen, 2014 ▸) for structures containing imidazole or imidazolium rings with nitrate anions returned 21 hits. Mol­ecular chains with bifurcated hydrogen bonds between imidazol-3-ium cations and nitrate anions as found in (II) have been reported for 2-(1-naphthyl­diazen­yl)-1*H*-imidazol-3-ium nitrate (Pramanik *et al.*, 2010 ▸), 2-azido­imidazolium nitrate (Tang *et al.*, 2012 ▸) and 2-phenyl­imidazolium nitrate hemihydrate (Zhang *et al.*, 2007 ▸). Mol­ecular chains similar to those observed in (I) with pairs of imidazole and imidazolium rings linked through bifurcated hydrogen bonds to nitrate anions are also found in the structure of 2-(1*H*-imidazol-2-yl)-1*H*-imidazol-3-ium nitrate (Jin *et al.*, 2011 ▸).

## Synthesis and crystallization   

All chemicals were purchased from Aldrich (Germany) and were used as received. Single crystals suitable for X-ray studies of (II) were first obtained by serendipity when a mixture of 2-methyl­imidazole and concentrated nitric acid was added to tri­methyl­tin acetate in methanol. Colourless single crystals of (I) were obtained after slow evaporation at room temperature of an aqueous solution consisting of 2-methyl­imidazole and concentrated nitric acid in a 2:1 ratio. Compound (II) can also be prepared in a similar way by changing the ratio between 2-methyl­imidazole and nitric acid to 1:1.

## Refinement   

Crystal data, data collection and structure refinement details are summarized in Table 3 ▸. For (I), all H atoms were clearly discernible from difference Fourier maps and were freely refined. Half-occupancy of H1 is required for structural reasons and was indicated by the values of the residual density peaks found in the difference Fourier map (0.83 *vs* 0.47 e Å^−3^ for an occupancy factor of 1 and 0.5, respectively). For (II), the H atoms bound to C were placed in calculated positions and then refined using a riding model with C—H = 0.95 Å (aromatic) and 0.98 Å (meth­yl) and *U*_iso_(H) = 1.2 and 1.5*U*_eq_(C), respectively. As a result of the mirror symmetry of the 2-methyl-1*H*-imidazol-3-ium cation, the methyl H atoms are statistically disordered over two positions. H atoms bound to N atoms were located from a difference Fourier map and were freely refined.

## Supplementary Material

Crystal structure: contains datablock(s) global, II, I. DOI: 10.1107/S2056989016003789/wm5276sup1.cif

Structure factors: contains datablock(s) I. DOI: 10.1107/S2056989016003789/wm5276Isup2.hkl

Structure factors: contains datablock(s) II. DOI: 10.1107/S2056989016003789/wm5276IIsup3.hkl

Supporting information file. DOI: 10.1107/S2056989016003789/wm5276Isup4.cml

Supporting information file. DOI: 10.1107/S2056989016003789/wm5276IIsup5.cml

CCDC references: 1458009, 1458008

Additional supporting information:  crystallographic information; 3D view; checkCIF report

## Figures and Tables

**Table 1:** Hydrogen-bond geometry (Å, °) for (I)

*D*—H⋯*A*	*D*—H	H⋯*A*	*D*⋯*A*	*D*—H⋯*A*
N2—H2⋯O1^i^	0.845 (19)	2.594 (19)	3.1837 (14)	127.9 (15)
N2—H2⋯O2	0.845 (19)	2.06 (2)	2.9031 (10)	172.5 (18)
N1—H1⋯N1^ii^	0.83 (3)	1.86 (3)	2.678 (2)	173 (4)

Symmetry codes: (i) 
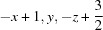
; (ii) 
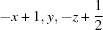
.

**Table 2:** Hydrogen-bond geometry (Å, °) for (II)

*D*—H⋯*A*	*D*—H	H⋯*A*	*D*⋯*A*	*D*—H⋯*A*
N1—H1⋯O1^i^	0.82 (4)	2.12 (4)	2.894 (2)	157 (4)
N1—H1⋯O3^i^	0.82 (4)	2.41 (4)	3.125 (3)	147 (4)
N2—H2⋯O1	0.94 (3)	1.83 (3)	2.760 (2)	167 (3)
N2—H2⋯O2	0.94 (3)	2.50 (3)	3.231 (2)	135 (2)

Symmetry code: (i) 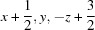.

**Table 3:** Experimental details

	(I)	(II)
Crystal data		
Chemical formula	C_4_H_6_N_2_^+^·NO_3_^−^·C_4_H_7_N_2_	C_4_H_7_N^2+^·NO_3_^−^
*M*_r_	227.23	145.13
Crystal system, space group	Monoclinic, *C*2/*c*	Orthorhombic, *P* *n* *m* *a*
Temperature (K)	100	110
*a*, *b*, *c* (Å)	10.1879 (4), 10.0912 (4), 11.9055 (5)	14.1402 (11), 6.2297 (5), 7.4571 (6)
α, β, γ (°)	90, 115.188 (2), 90	90, 90, 90
*V* (Å^3^)	1107.60 (8)	656.89 (9)
*Z*	4	4
Radiation type	Ga *K*α, λ = 1.34139 Å	Ga *K*α, λ = 1.34139 Å
μ (mm^−1^)	0.58	0.70
Crystal size (mm)	0.25 × 0.19 × 0.19	0.09 × 0.04 × 0.03

Data collection		
Diffractometer	Bruker Venture Metaljet	Bruker Venture Metaljet
Absorption correction	Multi-scan (*SADABS*; Krause *et al.*, 2015 ▸)	Multi-scan (*SADABS*; Krause *et al.*, 2015 ▸)
*T*_min_, *T*_max_	0.682, 0.752	0.471, 0.752
No. of measured, independent and observed [*I* > 2σ(*I*)] reflections	8771, 1286, 1210	13253, 817, 761
*R*_int_	0.033	0.060
(sin θ/λ)_max_ (Å^−1^)	0.650	0.651

Refinement		
*R*[*F* ^2^ > 2σ(*F* ^2^)], *wR*(*F* ^2^), *S*	0.034, 0.100, 1.04	0.049, 0.146, 1.04
No. of reflections	1286	817
No. of parameters	102	68
H-atom treatment	All H-atom parameters refined	H atoms treated by a mixture of independent and constrained refinement
Δρ_max_, Δρ_min_ (e Å^−3^)	0.25, −0.20	0.22, −0.28

Computer programs: *APEX2* and, *SAINT* (Bruker, 2014 ▸), *SHELXT* (Sheldrick, 2015*a* ▸), *SHELXL2014* (Sheldrick, 2015*b* ▸), *OLEX2* (Dolomanov *et al.*, 2009 ▸), *Mercury* (Macrae *et al.*, 2008 ▸), and *publCIF* (Westrip, 2010 ▸).

## supplementary crystallographic information

### (I) 2-Methyl-1H-imidazol-3-ium nitrate–2-methyl-1H-imidazole (1/1) Crystal data

**Table:**

C_4_H_6_N_2_^+^·NO_3_^−^·C_4_H_7_N_2_	*F*(000) = 480
*M**_r_* = 227.23	*D*_x_ = 1.363 Mg m^−^^3^
Monoclinic, *C*2/*c*	Ga *K*α radiation, λ = 1.34139 Å
*a* = 10.1879 (4) Å	Cell parameters from 6125 reflections
*b* = 10.0912 (4) Å	θ = 5.7–60.7°
*c* = 11.9055 (5) Å	µ = 0.58 mm^−^^1^
β = 115.188 (2)°	*T* = 100 K
*V* = 1107.60 (8) Å^3^	Block, clear light colourless
*Z* = 4	0.25 × 0.19 × 0.19 mm

### (I) 2-Methyl-1H-imidazol-3-ium nitrate–2-methyl-1H-imidazole (1/1) Data collection

**Table:**

Bruker Venture Metaljet diffractometer	1286 independent reflections
Radiation source: Metal Jet, Gallium Liquid Metal Jet Source	1210 reflections with *I* > 2σ(*I*)
Helios MX Mirror Optics monochromator	*R*_int_ = 0.033
Detector resolution: 10.24 pixels mm^-1^	θ_max_ = 60.7°, θ_min_ = 5.7°
ω and φ scans	*h* = −12→13
Absorption correction: multi-scan (SADABS; Krause *et al.*, 2015)	*k* = −13→13
*T*_min_ = 0.682, *T*_max_ = 0.752	*l* = −15→13
8771 measured reflections	

### (I) 2-Methyl-1H-imidazol-3-ium nitrate–2-methyl-1H-imidazole (1/1) Refinement

**Table:**

Refinement on *F*^2^	Primary atom site location: structure-invariant direct methods
Least-squares matrix: full	Hydrogen site location: difference Fourier map
*R*[*F*^2^ > 2σ(*F*^2^)] = 0.034	All H-atom parameters refined
*wR*(*F*^2^) = 0.100	*w* = 1/[σ^2^(*F*_o_^2^) + (0.053*P*)^2^ + 0.953*P*] where *P* = (*F*_o_^2^ + 2*F*_c_^2^)/3
*S* = 1.04	(Δ/σ)_max_ < 0.001
1286 reflections	Δρ_max_ = 0.25 e Å^−^^3^
102 parameters	Δρ_min_ = −0.20 e Å^−^^3^
0 restraints	

### (I) 2-Methyl-1H-imidazol-3-ium nitrate–2-methyl-1H-imidazole (1/1) Special details

**Table:**

Experimental. X-ray crystallographic data for I were collected from a single crystal sample, which was mounted on a loop fiber. Data were collected using a Bruker Venture diffractometer equipped with a Photon 100 CMOS Detector, a Helios MX optics and a Kappa goniometer. The crystal-to-detector distance was 4.0 cm, and the data collection was carried out in 1024 x 1024 pixel mode.
Geometry. All esds (except the esd in the dihedral angle between two l.s. planes) are estimated using the full covariance matrix. The cell esds are taken into account individually in the estimation of esds in distances, angles and torsion angles; correlations between esds in cell parameters are only used when they are defined by crystal symmetry. An approximate (isotropic) treatment of cell esds is used for estimating esds involving l.s. planes.

### (I) 2-Methyl-1H-imidazol-3-ium nitrate–2-methyl-1H-imidazole (1/1) Fractional atomic coordinates and isotropic or equivalent isotropic displacement parameters (Å^2^)

**Table:**

	*x*	*y*	*z*	*U*_iso_*/*U*_eq_	Occ. (<1)
N1	0.56964 (11)	0.62468 (10)	0.37404 (10)	0.0219 (3)	
H1	0.533 (4)	0.626 (3)	0.297 (3)	0.034 (9)*	0.5
N2	0.59828 (11)	0.65353 (10)	0.56421 (9)	0.0198 (2)	
C1	0.37127 (14)	0.74799 (14)	0.39888 (12)	0.0265 (3)	
H1A	0.386 (2)	0.837 (2)	0.3796 (19)	0.053 (6)*	
H1B	0.303 (2)	0.713 (2)	0.3230 (19)	0.049 (5)*	
H1C	0.333 (2)	0.741 (2)	0.454 (2)	0.058 (6)*	
C2	0.51030 (12)	0.67449 (11)	0.44456 (10)	0.0189 (3)	
H2	0.578 (2)	0.6791 (19)	0.6223 (17)	0.036 (4)*	
C3	0.70215 (14)	0.57154 (12)	0.45264 (12)	0.0251 (3)	
H3	0.766 (2)	0.5300 (18)	0.4208 (15)	0.037 (4)*	
C4	0.72067 (13)	0.58907 (12)	0.57091 (12)	0.0238 (3)	
H4	0.802 (2)	0.5656 (17)	0.6485 (16)	0.034 (4)*	
O1	0.45994 (10)	0.92001 (9)	0.82112 (8)	0.0274 (2)	
O2	0.5000	0.73329 (12)	0.7500	0.0229 (3)	
N3	0.5000	0.85988 (14)	0.7500	0.0194 (3)	

### (I) 2-Methyl-1H-imidazol-3-ium nitrate–2-methyl-1H-imidazole (1/1) Atomic displacement parameters (Å^2^)

**Table:**

	*U*^11^	*U*^22^	*U*^33^	*U*^12^	*U*^13^	*U*^23^
N1	0.0253 (5)	0.0217 (5)	0.0215 (5)	0.0005 (4)	0.0127 (4)	0.0000 (4)
N2	0.0216 (5)	0.0213 (5)	0.0177 (5)	0.0016 (4)	0.0094 (4)	0.0007 (4)
C1	0.0218 (6)	0.0317 (7)	0.0260 (6)	0.0055 (5)	0.0100 (5)	0.0035 (5)
C2	0.0198 (5)	0.0183 (5)	0.0196 (5)	−0.0012 (4)	0.0094 (4)	0.0005 (4)
C3	0.0253 (6)	0.0228 (6)	0.0315 (6)	0.0045 (5)	0.0164 (5)	0.0010 (5)
C4	0.0216 (6)	0.0215 (6)	0.0269 (6)	0.0036 (4)	0.0089 (5)	0.0029 (4)
O1	0.0335 (5)	0.0278 (5)	0.0261 (5)	0.0028 (4)	0.0176 (4)	−0.0026 (3)
O2	0.0258 (6)	0.0211 (6)	0.0238 (6)	0.000	0.0123 (5)	0.000
N3	0.0164 (6)	0.0234 (7)	0.0170 (6)	0.000	0.0056 (5)	0.000

### (I) 2-Methyl-1H-imidazol-3-ium nitrate–2-methyl-1H-imidazole (1/1) Geometric parameters (Å, º)

**Table:**

N1—H1	0.83 (3)	C1—H1C	0.90 (2)
N1—C2	1.3247 (15)	C1—C2	1.4821 (16)
N1—C3	1.3822 (17)	C3—H3	0.974 (19)
N2—C2	1.3381 (15)	C3—C4	1.3504 (18)
N2—H2	0.845 (19)	C4—H4	0.971 (18)
N2—C4	1.3783 (16)	O1—N3	1.2433 (11)
C1—H1A	0.95 (2)	O2—N3	1.2774 (19)
C1—H1B	0.94 (2)	N3—O1^i^	1.2433 (11)
			
C2—N1—H1	125 (3)	N1—C2—N2	109.58 (10)
C2—N1—C3	107.21 (10)	N1—C2—C1	125.45 (11)
C3—N1—H1	128 (3)	N2—C2—C1	124.91 (11)
C2—N2—H2	122.4 (13)	N1—C3—H3	121.6 (10)
C2—N2—C4	108.41 (10)	C4—C3—N1	108.53 (11)
C4—N2—H2	129.1 (13)	C4—C3—H3	129.9 (10)
H1A—C1—H1B	104.5 (17)	N2—C4—H4	123.6 (10)
H1A—C1—H1C	114.3 (18)	C3—C4—N2	106.26 (11)
H1B—C1—H1C	107.4 (19)	C3—C4—H4	130.1 (10)
C2—C1—H1A	109.7 (13)	O1—N3—O1^i^	121.58 (14)
C2—C1—H1B	111.1 (12)	O1^i^—N3—O2	119.21 (7)
C2—C1—H1C	109.7 (14)	O1—N3—O2	119.21 (7)
			
N1—C3—C4—N2	−0.02 (14)	C3—N1—C2—C1	176.33 (12)
C2—N1—C3—C4	0.57 (14)	C4—N2—C2—N1	0.92 (13)
C2—N2—C4—C3	−0.53 (14)	C4—N2—C2—C1	−176.35 (11)
C3—N1—C2—N2	−0.91 (13)		

Symmetry code: (i) −*x*+1, *y*, −*z*+3/2.

### (I) 2-Methyl-1H-imidazol-3-ium nitrate–2-methyl-1H-imidazole (1/1) Hydrogen-bond geometry (Å, º)

**Table:**

*D*—H···*A*	*D*—H	H···*A*	*D*···*A*	*D*—H···*A*
N2—H2···O1^i^	0.845 (19)	2.594 (19)	3.1837 (14)	127.9 (15)
N2—H2···O2	0.845 (19)	2.06 (2)	2.9031 (10)	172.5 (18)
N1—H1···N1^ii^	0.83 (3)	1.86 (3)	2.678 (2)	173 (4)

Symmetry codes: (i) −*x*+1, *y*, −*z*+3/2; (ii) −*x*+1, *y*, −*z*+1/2.

### (II) 2-Methyl-1H-imidazol-3-ium nitrate Crystal data

**Table:**

C_4_H_7_N^2+^·NO_3_^−^	*D*_x_ = 1.467 Mg m^−^^3^
*M**_r_* = 145.13	Ga *K*α radiation, λ = 1.34139 Å
Orthorhombic, *P**n**m**a*	Cell parameters from 9976 reflections
*a* = 14.1402 (11) Å	θ = 5.2–60.7°
*b* = 6.2297 (5) Å	µ = 0.70 mm^−^^1^
*c* = 7.4571 (6) Å	*T* = 110 K
*V* = 656.89 (9) Å^3^	Block, clear light colorless
*Z* = 4	0.09 × 0.04 × 0.03 mm
*F*(000) = 304	

### (II) 2-Methyl-1H-imidazol-3-ium nitrate Data collection

**Table:**

Bruker Venture Metaljet diffractometer	817 independent reflections
Radiation source: Metal Jet, Gallium Liquid Metal Jet Source	761 reflections with *I* > 2σ(*I*)
Helios MX Mirror Optics monochromator	*R*_int_ = 0.060
Detector resolution: 10.24 pixels mm^-1^	θ_max_ = 60.8°, θ_min_ = 8.1°
ω and φ scans	*h* = −16→18
Absorption correction: multi-scan (SADABS; Krause *et al.*, 2015)	*k* = −8→8
*T*_min_ = 0.471, *T*_max_ = 0.752	*l* = −9→9
13253 measured reflections	

### (II) 2-Methyl-1H-imidazol-3-ium nitrate Refinement

**Table:**

Refinement on *F*^2^	0 restraints
Least-squares matrix: full	Hydrogen site location: mixed
*R*[*F*^2^ > 2σ(*F*^2^)] = 0.049	H atoms treated by a mixture of independent and constrained refinement
*wR*(*F*^2^) = 0.146	*w* = 1/[σ^2^(*F*_o_^2^) + (0.0855*P*)^2^ + 0.2528*P*] where *P* = (*F*_o_^2^ + 2*F*_c_^2^)/3
*S* = 1.04	(Δ/σ)_max_ < 0.001
817 reflections	Δρ_max_ = 0.22 e Å^−^^3^
68 parameters	Δρ_min_ = −0.28 e Å^−^^3^

### (II) 2-Methyl-1H-imidazol-3-ium nitrate Special details

**Table:**

Experimental. X-ray crystallographic data for I were collected from a single crystal sample, which was mounted on a loop fiber. Data were collected using a Bruker Venture diffractometer equipped with a Photon 100 CMOS Detector, a Helios MX optics and a Kappa goniometer. The crystal-to-detector distance was 4.0 cm, and the data collection was carried out in 1024 x 1024 pixel mode.
Geometry. All esds (except the esd in the dihedral angle between two l.s. planes) are estimated using the full covariance matrix. The cell esds are taken into account individually in the estimation of esds in distances, angles and torsion angles; correlations between esds in cell parameters are only used when they are defined by crystal symmetry. An approximate (isotropic) treatment of cell esds is used for estimating esds involving l.s. planes.

### (II) 2-Methyl-1H-imidazol-3-ium nitrate Fractional atomic coordinates and isotropic or equivalent isotropic displacement parameters (Å^2^)

**Table:**

	*x*	*y*	*z*	*U*_iso_*/*U*_eq_	Occ. (<1)
N1	0.75103 (14)	0.2500	0.7473 (3)	0.0489 (5)	
H1	0.791 (3)	0.2500	0.828 (5)	0.104 (14)*	
N2	0.62239 (12)	0.2500	0.5968 (2)	0.0383 (5)	
H2	0.558 (2)	0.2500	0.566 (4)	0.065 (9)*	
C1	0.6012 (2)	0.2500	0.9290 (3)	0.0581 (7)	
H1A	0.6415	0.2943	1.0295	0.087*	0.5
H1B	0.5768	0.1053	0.9515	0.087*	0.5
H1C	0.5483	0.3504	0.9170	0.087*	0.5
C2	0.65719 (16)	0.2500	0.7623 (3)	0.0409 (5)	
C3	0.77534 (16)	0.2500	0.5686 (3)	0.0493 (6)	
H3	0.8376	0.2500	0.5208	0.059*	
C4	0.69503 (16)	0.2500	0.4755 (3)	0.0448 (6)	
H4	0.6892	0.2500	0.3486	0.054*	
O1	0.42825 (10)	0.2500	0.55858 (18)	0.0449 (5)	
O2	0.46812 (12)	0.2500	0.2772 (2)	0.0498 (5)	
O3	0.32003 (10)	0.2500	0.3546 (2)	0.0482 (5)	
N3	0.40563 (11)	0.2500	0.3932 (2)	0.0384 (5)	

### (II) 2-Methyl-1H-imidazol-3-ium nitrate Atomic displacement parameters (Å^2^)

**Table:**

	*U*^11^	*U*^22^	*U*^33^	*U*^12^	*U*^13^	*U*^23^
N1	0.0421 (10)	0.0503 (11)	0.0542 (11)	0.000	−0.0184 (8)	0.000
N2	0.0319 (8)	0.0507 (10)	0.0324 (8)	0.000	−0.0023 (6)	0.000
C1	0.0775 (18)	0.0625 (15)	0.0342 (11)	0.000	0.0076 (10)	0.000
C2	0.0430 (11)	0.0460 (11)	0.0337 (10)	0.000	−0.0045 (8)	0.000
C3	0.0357 (11)	0.0509 (13)	0.0612 (14)	0.000	0.0057 (9)	0.000
C4	0.0448 (12)	0.0522 (12)	0.0374 (10)	0.000	0.0064 (8)	0.000
O1	0.0360 (8)	0.0681 (10)	0.0308 (7)	0.000	−0.0011 (5)	0.000
O2	0.0449 (9)	0.0686 (11)	0.0359 (8)	0.000	0.0092 (6)	0.000
O3	0.0348 (7)	0.0592 (10)	0.0507 (9)	0.000	−0.0103 (6)	0.000
N3	0.0344 (8)	0.0485 (10)	0.0323 (8)	0.000	−0.0005 (6)	0.000

### (II) 2-Methyl-1H-imidazol-3-ium nitrate Geometric parameters (Å, º)

**Table:**

N1—H1	0.82 (4)	C1—H1C	0.9800
N1—C2	1.332 (3)	C1—C2	1.474 (3)
N1—C3	1.376 (3)	C3—H3	0.9500
N2—H2	0.94 (3)	C3—C4	1.331 (3)
N2—C2	1.329 (2)	C4—H4	0.9500
N2—C4	1.369 (3)	O1—N3	1.274 (2)
C1—H1A	0.9800	O2—N3	1.237 (2)
C1—H1B	0.9800	O3—N3	1.244 (2)
			
C2—N1—H1	128 (3)	N1—C2—C1	127.3 (2)
C2—N1—C3	109.29 (19)	N2—C2—N1	106.91 (18)
C3—N1—H1	122 (3)	N2—C2—C1	125.8 (2)
C2—N2—H2	126.1 (18)	N1—C3—H3	126.5
C2—N2—C4	109.63 (18)	C4—C3—N1	106.98 (19)
C4—N2—H2	124.3 (18)	C4—C3—H3	126.5
H1A—C1—H1B	109.5	N2—C4—H4	126.4
H1A—C1—H1C	109.5	C3—C4—N2	107.19 (19)
H1B—C1—H1C	109.5	C3—C4—H4	126.4
C2—C1—H1A	109.5	O2—N3—O1	119.85 (17)
C2—C1—H1B	109.5	O2—N3—O3	122.22 (18)
C2—C1—H1C	109.5	O3—N3—O1	117.93 (16)
			
N1—C3—C4—N2	0.000 (1)	C3—N1—C2—C1	180.000 (1)
C2—N1—C3—C4	0.000 (1)	C4—N2—C2—N1	0.000 (1)
C2—N2—C4—C3	0.000 (1)	C4—N2—C2—C1	180.000 (1)
C3—N1—C2—N2	0.000 (1)		

### (II) 2-Methyl-1H-imidazol-3-ium nitrate Hydrogen-bond geometry (Å, º)

**Table:**

*D*—H···*A*	*D*—H	H···*A*	*D*···*A*	*D*—H···*A*
N1—H1···O1^i^	0.82 (4)	2.12 (4)	2.894 (2)	157 (4)
N1—H1···O3^i^	0.82 (4)	2.41 (4)	3.125 (3)	147 (4)
N2—H2···O1	0.94 (3)	1.83 (3)	2.760 (2)	167 (3)
N2—H2···O2	0.94 (3)	2.50 (3)	3.231 (2)	135 (2)

Symmetry code: (i) *x*+1/2, *y*, −*z*+3/2.
